# Local charge homogenization strategy enables ultra-high voltage tolerance of polyether electrolytes for 4.7 V lithium metal batteries

**DOI:** 10.1093/nsr/nwae436

**Published:** 2024-12-03

**Authors:** Yuanlong Wu, Piao Luo, Kexin Su, Mao Yu, Xin Song, Lianzhan Huang, Shaocong Zhang, Huiyu Song, Li Du, Weishu Liu, Zhiming Cui

**Affiliations:** Guangdong Provincial Key Laboratory of Fuel Cell Technology, School of Chemistry and Chemical Engineering, South China University of Technology, Guangzhou 510641, China; Guangdong Provincial Key Laboratory of Fuel Cell Technology, School of Chemistry and Chemical Engineering, South China University of Technology, Guangzhou 510641, China; Guangdong Provincial Key Laboratory of Fuel Cell Technology, School of Chemistry and Chemical Engineering, South China University of Technology, Guangzhou 510641, China; Department of Materials Science and Engineering, Southern University of Science and Technology, Shenzhen 518055, China; Guangdong Provincial Key Laboratory of Advanced Energy Storage Materials, School of Materials Science and Engineering, South China University of Technology, Guangzhou 510641, China; Guangdong Provincial Key Laboratory of Fuel Cell Technology, School of Chemistry and Chemical Engineering, South China University of Technology, Guangzhou 510641, China; School of Software Engineering, South China University of Technology, Guangzhou 510006, China; Guangdong Provincial Key Laboratory of Fuel Cell Technology, School of Chemistry and Chemical Engineering, South China University of Technology, Guangzhou 510641, China; Guangdong Provincial Key Laboratory of Fuel Cell Technology, School of Chemistry and Chemical Engineering, South China University of Technology, Guangzhou 510641, China; Department of Materials Science and Engineering, Southern University of Science and Technology, Shenzhen 518055, China; Guangdong Provincial Key Laboratory of Fuel Cell Technology, School of Chemistry and Chemical Engineering, South China University of Technology, Guangzhou 510641, China

**Keywords:** lithium metal batteries, solid polymer electrolytes, *in-situ* polymerization, high voltage, local charge homogenization

## Abstract

*In-situ* fabricated polyether electrolytes have been regarded as one of the most promising solid electrolyte systems. Nevertheless, they cannot match high-voltage cathodes over 4.3 V due to their poor oxidative stability. Herein, we propose an effective local charge homogenization strategy based on the triglycidyl isocyanurate (TGIC) crosslinker, achieving ultra-high-voltage electrochemical stability of polyether electrolytes (viz. PTIDOL) at cutoff voltages up to 4.7 V. The introduction of TGIC optimizes the Li^+^ solvation environment, thereby homogenizing the charge distribution at ether oxygen (EO) sites, resulting in significantly enhanced oxidative stability of the polyether main chain. Consequently, the Li|PTIDOL|LiNi_0.6_Co_0.2_Mn_0.2_O_2_ (NCM622) cell achieves long-term operation at an ultra-high cutoff voltage with a capacity retention of 81.8% after 400 cycles, one of the best results reported for polyether electrolytes to date. This work provides significant insights for the development of polyether electrolytes with high-voltage tolerance and the advancement of high-energy-density batteries.

## Teaser

By developing a local charge homogenization strategy, this work reinforces the high-voltage tolerance of polyether electrolytes in a way that differs from conventional strategies.

## INTRODUCTION

The rapid development of electric vehicles and portable electronic devices has stimulated intense research on solid-state lithium metal batteries (SLMBs) [1–6]. Solid-state polymer electrolytes (SPEs) based on *in-situ* polymerization are considered the most promising solid-state electrolytes (SSEs) due to their ability to achieve intimate interface contact and good compatibility with current battery assembly lines [7–9]. Generally, *in-situ* polymerized electrolytes can be mainly divided into two categories: polyester electrolytes and polyether electrolytes [10]. Compared to polyester electrolytes, polyether electrolytes are widely studied because of their comprehensive advantages, including high ionic conductivity and excellent compatibility with lithium metal anodes [11–14]. However, their poor oxidative stability (<4 V) hinders the compatibility of polyether electrolytes with high-voltage cathodes, such as nickel-rich layered oxide cathodes like LiNi_1 − x − y_Co_x_Mn_y_O_2_ (NCM: 1 − x − y ≥ 0.6) [15,16].

It is well-established that terminal hydroxy (−OH) groups of polyether chains are susceptible to oxidation at high voltages (>4 V), resulting in the degradation of polyether electrolytes [17,18]. Accordingly, some strategies have been proposed to eliminate the terminal −OH groups in polyether electrolytes, such as functional group substitution [18,19] and crosslinked network construction [20–22]. For example, Yang *et al.* [19] substituted terminal −OH groups of poly(ethylene glycol) (PEG) with more stable groups (−OCH_3_) to obtain a stable polyether electrolyte that has an electrochemical stability window (ESW) to 4.3 V. Deng *et al.* [22] constructed a crosslinked copolymer network by using trimethylolpropane triglycidyl ether (TTE) as a crosslinker to eliminate terminal −OH groups, achieving the stable operation of Li||LiMn_0.8_Fe_0.2_PO_4_ (LMFP) batteries at 4.2 V. Notably, although many attempts have been made to eliminate the terminal −OH groups to enhance the oxidative stability of polyether electrolytes, it remains a great challenge for polyether electrolytes to operate at a cutoff voltage above 4.3 V. The unsatisfactory results imply that there are other intrinsic factors affecting the oxidative stability of polyether electrolytes. Recently, Sun *et al.* [19] conducted a detailed study on the oxidative stability of polyether electrolytes under high voltage and proposed a possible oxidation mechanism, i.e. the terminal −OH is oxidized in a voltage window of 4.05–4.3 V, while the ether chain (C−O−C) is oxidized at a voltage to over 4.3 V. The aforementioned progress inspires us to explore novel and practical strategies that enhance the oxidative stability of the polyether main chain, enabling polyether electrolytes to operate at cutoff voltages above 4.3 V.

In this contribution, we propose a new and feasible triglycidyl isocyanurate (TGIC)-based local charge homogenization strategy that can address the long-standing issue of the high-voltage tolerance (over 4.3 V) of polyether electrolytes. Theoretical calculations and characterizations elucidate the intrinsic mechanism of the local charge homogenization strategy for enhancing the oxidation stability of polyether electrolytes. The designed polyether electrolyte, PTIDOL, exhibits high oxidative stability and excellent Li^+^ transport kinetics, demonstrating a wide ESW of 5.1 V and a high Li^+^ transference number(t_Li+_) of 0.82. Due to the inhibition of electrolyte decomposition at high voltage, it is conducive to the formation of inorganic-rich cathode-electrolyte interphase (CEI), which greatly improves the compatibility between polyether electrolytes and high voltage cathodes. The Li|PTIDOL|NCM622 cell delivers outstanding cycling stability at an ultra-high cutoff voltage of 4.7 V, paving the way for practical applications of high-voltage solid-state batteries.

## RESULTS AND DISCUSSION

### Principle of the local charge homogenization strategy and fabrication of PTIDOL

**Figure 1.**
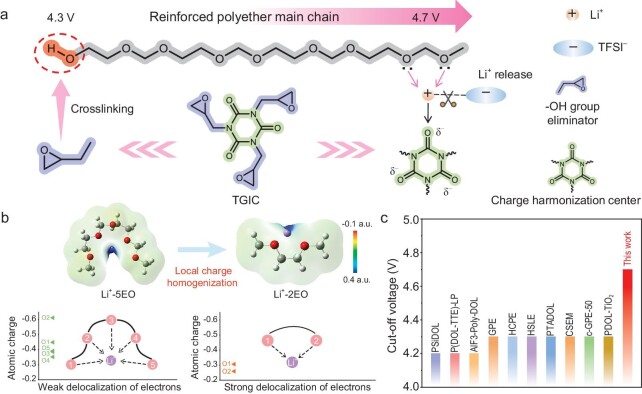
Design rationale and high voltage tolerance of PTIDOL. (a) Schematic illustration of the mechanism of the local charge homogenization strategy. (b) The calculated electrostatic potential of Li^+^-2EO and Li^+^-5EO and the atomic charge of EO. (c) Comparison of the cutoff voltage of PTIDOL and other reported polyether electrolytes.

It is known that eliminating terminal −OH groups can increase the cutoff voltage of polyether electrolyte batteries to 4.3 V. However, the inferior oxidative stability of the polyether main chain hinders their operation at voltages above 4.3 V, due to the oxidative decomposition resulting from the presence of unstable lone-pair electrons in the ether oxygen (EO) [23]. Herein, a localized charge homogenization strategy based on TGIC is developed to enhance the oxidative stability of the polyether main chain. As shown in Fig. 1a, the introduction of TGIC tailors the Li^+^ solvation structure due to abundant active sites at the crosslinker center, thereby increasing the Li^+^/EO ratio. The charge distribution of the EO on the polyether main chain exhibits significant delocalization from the EO linkage to Li^+^ as the Li^+^/EO ratio increases (Fig. 1b). Consequently, the highest occupied molecular orbital (HOMO) energy level of the Li^+^-polyether main chain complex shifts towards lower values, thereby enhancing the oxidative stability of the polyether electrolytes. As a conceptual validation, the designed polyether electrolyte operates stably at an ultra-high cutoff voltage of 4.7 V, which is rare in previous reports (Fig. 1c) [20–22,24–30].

**Figure 2.**
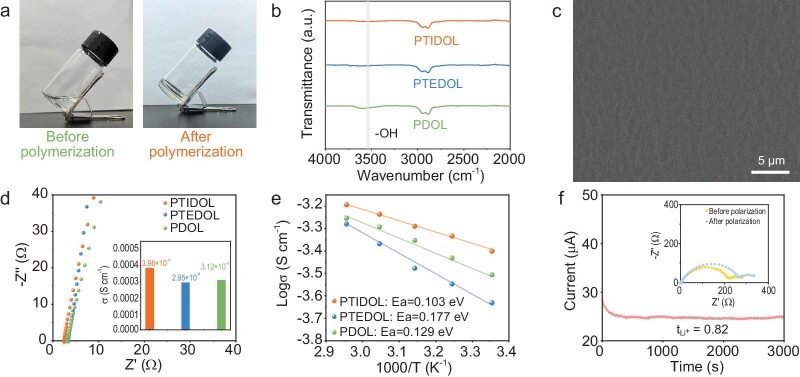
Physicochemical properties of the *in-situ* fabricated PTIDOL. (a) Optical images showing the *in-situ* solidification of liquid precursor to solid polymer electrolyte. (b) FTIR spectra of PTIDOL, PTEDOL and PDOL from 2000 cm^−1^ to 4000 cm^−1^. (c) Scanning electron microscopy (SEM) image of the PTIDOL membrane. (d) Nyquist curves of PTIDOL, PTEDOL and PDOL and corresponding ionic conductivity at room temperature. (e) Temperature-dependent ionic conductivities of the electrolytes and their fitting with Arrhenius models. (f) Chronoamperometry polarization curve and the impedance spectra before and after polarization of the Li||Li symmetric cell with PTIDOL.

PTIDOL is synthesized via copolymerization using 1,3-Dioxolane (DOL) as the monomer, TGIC as the crosslinker (5 wt% of DOL), and initiated by lithium difluoro(oxalato)borate (LiDFOB)-induced cationic ring-opening polymerization. The polymerization process is shown in Fig. S1. The end of TGIC is connected to the DOL chain by ring-opening of ethylene oxide; the three vertices of TGIC are connected to six DOL chains to form a unit structure, and the units are connected to form a huge 3D crosslinking network. For comparison, the polyether electrolyte (viz. PTEDOL) is prepared by copolymerization of DOL with the conventional three-arm crosslinker TTE, which lacks the charge harmonization center. Poly(1,3-Dioxolane) (PDOL) is synthesized by the homopolymerization of pure DOL. The optical images presented in Fig. 2a display the macroscopic transformation of the initial liquid precursor into a non-fluid solid electrolyte, providing evidence of the effective polymerization of PTIDOL. The ring-opening crosslinking polymerization of DOL and TGIC is confirmed by Fourier transform infrared (FTIR) spectroscopy. The characteristic epoxide band at 915 cm^−1^ disappears after crosslinking, as shown in Fig. S2, indicating that epoxides react to form the network structure [31]. A similar phenomenon is also observed in the polymerization of PTEDOL and PDOL. As seen in Fig. 2b, the −OH groups in PTIDOL and PTEDOL greatly decrease compared to PDOL, indicating that the terminal −OH groups of polyether are end-capped by ether rings from the crosslinker via copolymerization. Additionally, the resonance signal of DOL monomers is not observed in ^13^C solid-state nuclear magnetic resonance (NMR) spectroscopy of PTIDOL (Fig. S3). The results demonstrate that the introduction of TGIC enhances the monomer conversion of DOL. The NMR spectroscopy results of PTEDOL and PDOL are consistent with previous literature reports [8,22], indicating successful synthesis of the three polyether electrolytes. Figure 2c and Fig. S4 demonstrate the scanning electron microscopy (SEM) image of the original polyethylene (PE) separator and three polyether electrolytes polymerized within the separator. A SEM image based on the PTIDOL film surface shows a smoother and more uniform surface compared to the other two electrolyte films without obvious voids, suggesting the successful *in-situ* solidification of precursor solutions inside the PE frameworks. In SPEs, the absence of a crystallization region in the *in-situ* formed SPEs is conducive for the rapid migration of Li^+^, thereby resulting in the high ionic conductivity of electrolyte. X-ray diffraction (XRD) measurements are conducted to explore the crystallinity of the prepared polymers (Fig. S5). The three electrolytes show a broad diffusion peak between 10° and 30°, indicating a typical amorphous phase of polymer. The differential scanning calorimetry (DSC) tests of three polyether electrolytes are shown in Fig. S6, with the glass transition temperature (Tg) of each sample consistently remaining below −50°C. To confirm the feasibility of using these three types of polyether electrolytes in SLMBs, electrochemical impedance spectroscopy (EIS) is conducted at room temperature (Fig. 2d). PTIDOL exhibits a high ionic conductivity of 3.96 × 10^−4^ S cm^−1^, surpassing PTEDOL (2.95 × 10^−4^ S cm^−1^) and PDOL (3.12 × 10^−4^ S cm^−1^). Although the chain flexibility of PTIDOL is slightly restricted compared to PDOL based on DSC results, the higher free Li^+^ concentration in PTIDOL due to the abundant polar groups on TGIC that facilitate Li salt dissociation significantly improves the ionic conductivity of the PTIDOL electrolytes. Detailed discussion on the Li^+^ solvation environment will follow. Additionally, Fig. 2e illustrates the temperature dependence of ionic conductivity of the electrolytes within a temperature range of 25–65°C. When fitting the data using the Arrhenius equation, PTIDOL demonstrates a lower activation energy barrier of 0.103 eV compared to the other two electrolytes, facilitating the transport of Li^+^. PTIDOL manifests a higher t_Li+_ of 0.82 than PTEDOL (0.61) and PDOL (0.60) (Fig. 2f and Fig. S7), due to the lower transfer barrier for Li^+^ in PTIDOL. According to Sand's time model, a higher t_Li+_ can effectively reduce the concentration polarization of Li^+^, suppressing the growth of Li dendrites. Moreover, PTIDOL exhibited minimal electronic conductivity, providing a robust safeguard against the formation of lithium dendrites in the battery (Fig. S8).

### Li^+^ solvation environment and oxidative stability mechanism

**Figure 3.**
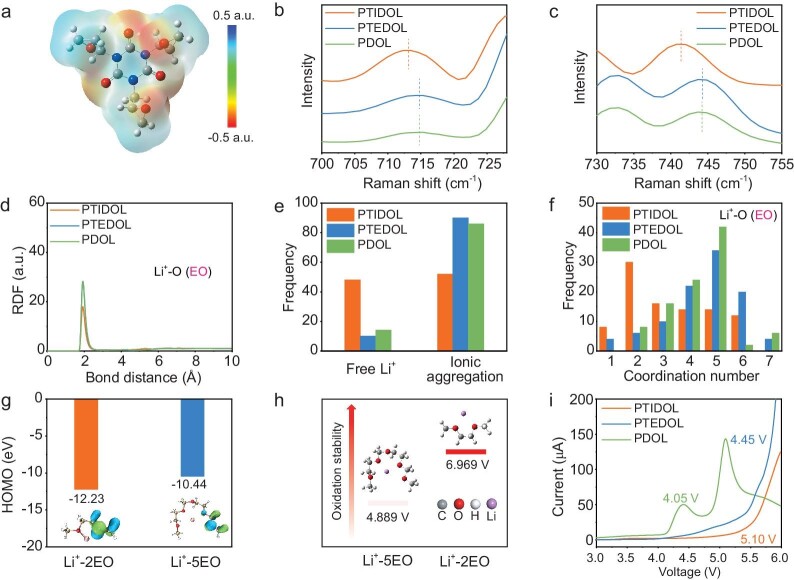
Li^+^ solvation environment and oxidative stability mechanism. (a) The calculated electrostatic potential of the TGIC. Color schemes are used: C, gray; N, blue; O, red; H, white. Raman spectra of PTIDOL, PTEDOL and PDOL from (b) 700 cm^−1^ to 728 cm^−1^ and (c) 730 cm^−1^ to 755 cm^−1^. (d) Calculated radial distribution functions of Li-O(EO) for PTIDOL, PTEDOL and PDOL. (e) Frequency distribution of free Li^+^ and ionic aggregation. (f) Coordination number distribution of Li^+^ with ether oxygen from polyether. (g) Calculated HOMO energy values of Li^+^-2EO and Li^+^-5EO. (h) The calculated oxidation potential of Li^+^-2EO and Li^+^-5EO. (i) Linear sweep voltammograms (LSVs) of the electrolytes at a scan rate of 1 mV s^−1^ in the voltage range of 3–6 V.

In SPEs, the coordinating environment of Li^+^ with the polymer chain and anions profoundly influences the ion transport and oxidative stability of the electrolyte [32]. Density functional theory (DFT) elucidates the subtle interactions between the TGIC crosslinker and Li^+^. The optimized geometries and electron cloud distribution of the TGIC structure are shown in Fig. 3a. The red regions representing negative electrostatic potential indicate abundant polar groups at the center of TGIC, suggesting that TGIC has the ability to regulate the solvation structure as a ‘harmonizer’ through ion–dipole interactions. The binding energies of Li^+^/TFSI^−^ and Li^+^/DFOB^−^ within TGIC are −4.35 eV and −3.38 eV, which are significantly lower than those without TGIC (Fig. S9). The low binding energy between Li^+^ and anions facilitates the dissociation of Li salts, resulting in the release of a greater number of free Li^+^. Classical molecular dynamics (MD) simulations are conducted to investigate the solvation structure in three electrolyte systems. The initial MD models for PTIDOL, PTEDOL and PDOL are shown in Fig. S10, respectively. Firstly, the radial distribution functions (RDFs) of the electrolyte are analyzed to elucidate the solvation structure of Li^+^ in different electrolyte systems. The intensity in Li-O (TFSI^−^) and Li-O (DFOB^−^) coordination of PTIDOL is 26.8 and 33.3, which is lower than the other two electrolyte systems (Fig. S11). This indicates an increased amount of free Li^+^ in the PTIDOL system. Raman spectroscopy provides further experimental insight into the dissociation behavior of Li salts. Peaks observed within the range of 700–720 cm^−1^ and 730–750 cm^−1^ in Fig. 3b and c, correspond to the ring-breathing vibration of the DFOB^−^ [33] and the S-N-S stretching vibration of the TFSI^−^ [34]. PTIDOL displays a red shift compared to PTEDOL and PDOL due to the decreased coordination between anions and Li^+^, which is consistent with the MD results. Furthermore, it is evident from Fig. 3d that the intensity in Li^+^-O (EO) coordination of PTIDOL is also weaker than that of PTEDOL and PDOL. The result can be attributed to the stretching of polymer chains by a greater number of free Li^+^. In contrast, compared to PTEDOL and PDOL, the intensity in Li^+^-O (TFSI^−^), Li^+^-O (DFOB^−^) and Li^+^-O (EO) coordination remains unchanged, suggesting that conventional three-armed crosslinkers lack the ability to regulate the solvation structure.

The coordination of Li^+^ with EO groups on polymer chains reduces the electron density of the EO, consequently decreasing the HOMO energy level and enhancing oxidative stability [17]. However, distinct coordination structures lead to varying degrees of electron delocalization. Further examination of the local coordination environment of Li^+^ is conducted by analyzing the probability distribution of oxygen atoms within its first solvation shell. Following the first peak in each RDF, the subsequent valleys are regarded as the cutoff distances (3 Å) for the first solvation shell [35]. As shown in Fig. 3e, free Li^+^ (not coordinated with anions) constitutes 48% in PTIDOL, compared to only 10% and 14% in PTEDOL and PDOL, respectively. The increased free Li^+^ in PTIDOL stretches the polyether chains, decreasing the EO/Li^+^ ratio. From the statistical results, Li^+^ predominantly coordinates with two EO atoms in PTIDOL, while it coordinates with five EO atoms in PTEDOL and PDOL (Fig. 3f). As shown in Fig. 1b, the electron cloud density distribution of the Li^+^-2EO coordination structure exhibits a more notable delocalization from the EO linkage to the Li^+^. By calculating the Becke atomic charges, it can be observed that the charge on the EO in the Li^+^-2EO (O2 and O5) coordination structure is significantly lower than Li^+^-5EO (O2, O5, O7, O10 and O12), demonstrating a more pronounced electron delocalization of the EO in Li^+^-2EO (Tables S2 and S3). Furthermore, the phenomenon of electronic delocalization can be reflected by the change of chemical shift of ^13^C and ^7^Li NMR. Compared to PTEDOL and PDOL, all the chemical shifts of EO carbon (C−O) in PTIDOL show an obvious increase owing to the reduced electronic density on EO [32] (Fig. S12). As shown in Fig. S13, the ^7^Li NMR spectroscopy of PTIDOL displays a more negative chemical shift than PTEDOL and PDOL. The upfield shift of PTIDOL demonstrates the increase in electron density around Li^+^, which results in a relatively strong shielding of Li^+^, indicating a stronger electronic transfer from EO to Li^+^. Figure 3g illustrates the HOMO energy levels of two coordination configurations: Li^+^-2EO and Li^+^-5EO. The HOMO energy level of Li^+^-2EO (−12.23 eV) is notably lower than that of Li^+^-5EO (−10.44 eV), suggesting that the polyether main chain of PTIDOL possesses superior oxidative stability compared to the other two polyether electrolytes. The trend suggests that the change in the HOMO energy level is induced by the charge delocalization at the molecular level. In order to understand the oxidation reaction mechanism of the electrolyte, the oxidation potentials of Li^+^-2EO coordination and Li^+^-5EO coordination are further calculated using DFT based on the reported calculation methods [36]. The Li^+^-5EO coordination exhibits the lowest oxidation potential (4.889 V), whereas the Li^+^-2EO coordination shows a higher oxidation potential (6.969 V) (Fig. 3h). The simulation and characterization results above prove that introducing the ‘harmonizer’ into PTIDOL shifts the coordination of Li^+^ with the polyether main chain from Li^+^-5EO to Li^+^-2EO. Compared to Li^+^-5EO coordination, the local charge density at EO sites is lower in Li^+^-2EO coordination, significantly enhancing the oxidative stability of the polyether main chain. The electrochemical stability of electrolytes is assessed through linear sweep voltammetry (LSV). As shown in Fig. 3i, PTIDOL exhibits a high oxidation potential up to 5.10 V, surpassing that of PTEDOL (4.45 V) and PDOL (4.05 V). Overall, the elimination of terminal −OH groups and the homogenization of charge distribution at the EO sites on the main chain are the intrinsic mechanisms for the improved oxidative stability of PTIDOL.

### Electrochemical performance of full cells

**Figure 4.**
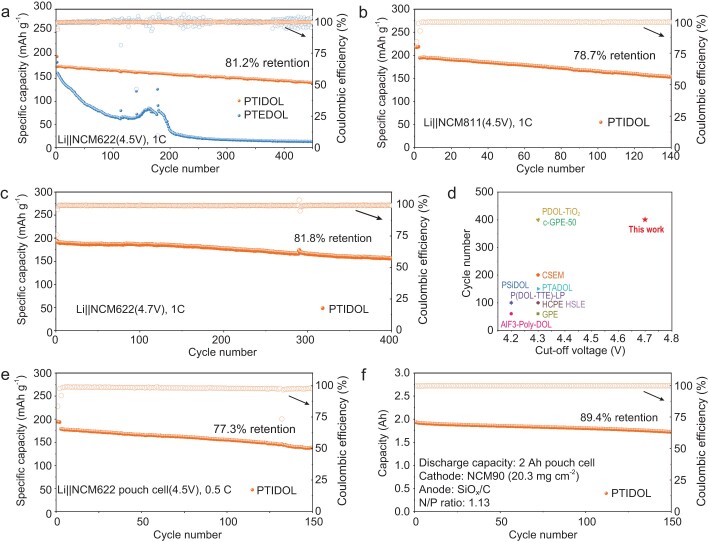
Electrochemical performance of full cells. (a) Cycling performance of Li|PTIDOL|NCM622 and Li|PTEDOL|NCM622 cells at 1 C and a charge cutoff voltage of 4.5 V. (b) Cycling performance of the Li|PTIDOL|NCM811 cell at 1 C and a charge cutoff voltage of 4.5 V. (c) Cycling performance of the Li|PTIDOL|NCM622 cell at 1 C and a charge cutoff voltage of 4.7 V. (d) Comparison of cycle numbers and cutoff voltages of PTIDOL and other reported polyether electrolytes. (e) Cycling performance of the Li|PTIDOL|NCM622 pouch cell at 0.5 C and a charge cutoff voltage of 4.5 V. (f) Cycling performance of the 2-Ah SiO_x_/C|PTIDOL|NCM90 pouch cell with a low N/P ratio of 1.13 (cathode mass loading: 20.3 mg cm^−2^).

PTIDOL, PTEDOL and PDOL are utilized to assemble Li||NCM622 cells, evaluating their cycle stability at high voltage. Initially, the performance of Li|PTEDOL|NCM622 and Li|PDOL|NCM622 cells is compared at a cutoff voltage of 4.3 V. As shown in Fig. S14, Li|PTEDOL|NCM622 retains 82.8% of its capacity after 120 cycles at 1 C. In contrast, the Li|PDOL|NCM622 cell retains only 60.9% of its capacity. The rapid capacity decay of the Li|PDOL|NCM622 cell is due to oxidative decomposition initiated by terminal −OH groups, and the resulting consumption of the electrolyte and the electrode. The performance of Li|PTIDOL|NCM622 and Li|PTEDOL|NCM622 cells, which eliminate terminal −OH groups, is tested at 4.5 V (Fig. 4a). The Li|PTIDOL|NCM622 cell operating at 4.5 V demonstrates an excellent long-cycling performance of 450 cycles with a high capacity retention of 81.2%. Furthermore, according to the charge-discharge profiles shown in Fig. S15, the cell using PTIDOL exhibits no obvious voltage polarization. Additionally, their interface resistance remains stable during cycling (Fig. S16), indicating excellent interface compatibility with both high-voltage cathodes and Li-metal anodes. In contrast, the capacity retention of the Li|PTEDOL|NCM622 cell deteriorates significantly, with an extremely low retention rate of only 7.9% after 450 cycles, and a severe increase in voltage polarization and interfacial resistance can also be observed (Figs S15 and S16). Compared to the Li|PTIDOL|NCM622 cell, the hysteresis voltage of the Li|PTEDOL|NCM622 cell rapidly deteriorates after the 150th cycle, indicating severe parasitic interfacial reactions during cycling (Fig. S17). The result indicates that eliminating terminal −OH groups alone cannot enable polyether electrolytes to operate stably at 4.5 V. Furthermore, the Li|PTIDOL|NCM811 cell can also operate stably at 1 C and a cutoff voltage of 4.5 V (Fig. 4b and Fig. S18). Additionally, the Li|PTIDOL|NCM622 cell is capable of delivering specific capacities of 183.2, 173.8, 159.2, 146.7 and 128.4 mAh g^−1^ at 0.5 C, 1 C, 2 C, 3 C and 5 C respectively, which are preferable to the Li|PTEDOL|NCM622 cell (Figs S19 and S20). It is noteworthy that the specific capacity values recovered to similar initial values when the current density turned to 0.5 C, indicating that the Li|PTIDOL|NCM622 cell exhibits superior rate performance and reversibility even at 4.5 V. To further investigate the electrochemical stability of the electrolyte at ultra-high cutoff voltage, the cycling stability of the Li|PTEDOL|NCM622 cell at 4.7 V cutoff voltage is tested. Benefitting from the elimination of terminal −OH groups and the enhanced oxidative stability of the polyether main chain, the Li|PTIDOL|NCM622 cell exhibits excellent cycling stability with a capacity retention rate of 81.8% after 400 cycles at 1 C (Fig. 4c and Fig. S21), which is unprecedented in previously reported studies (Fig. 4d) [20–22,24–30].

To further validate the potential of PTIDOL in practical applications, a Li|PTIDOL|NCM622 pouch cell is assembled and tested. As illustrated in Fig. 4e and Fig. S22, at a cutoff voltage of 4.5 V and a rate of 0.5 C, the capacity retention rate remained at 77.3% after 150 cycles. Even following a series of abuse tests, including bending, cutting and punching, the pouch cell could lighten an LED bulb. This demonstrates that PTIDOL promises excellent safety performance and showcases the promising application prospects of SLMBs (Fig. S23). Additionally, a pouch cell with a capacity of 2 Ah is manufactured using a SiO_x_/C anode and high-loading cathode (20.3 mg cm^−2^), with a low N/P ratio of 1.13. After cycling 150 cycles at 1 C rate, the pouch cell exhibits excellent cycling stability with a high capacity retention of 89.4% (Fig. 4f).

### CEI chemistry and structural evolution of NCM622

**Figure 5.**
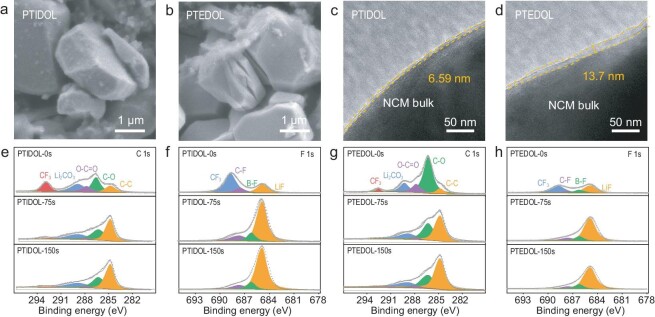
CEI chemistry and structural evolution of NCM622. (a and b) SEM morphologies of the NCM622 cathodes in (a) PTIDOL and (b) PTEDOL after 50 cycles. (c and d) HR-TEM images of the cathodes after 50 cycles in (c) PTIDOL and (d) PTEDOL. (e–h) XPS depth profiles of the NCM622 cathode harvested from the Li||NCM622 cell using (e and f) PTIDOL and (g and h) PTEDOL after 50 cycles at 1 C.

The improved oxidation stability of PTIDOL is further examined by studying the surface chemistry properties of the NCM622 cathodes after cycling. We characterize the cathode after cycling at 4.5 V using SEM and a high-resolution tunneling electron microscope (HR-TEM). Substantial disparities in surface morphology emerged on the cycled cathodes in PTIDOL and PTEDOL. As illustrated in Fig. 5a, the NCM622 particles cycled in PTIDOL display remarkably smooth surfaces and excellent structural integrity. In stark contrast, the NCM622 particles cycled in PTEDOL exhibit noticeable surface cracks (Fig. 5b). Typically, the cracking of Ni-rich cathodes stands as a pivotal factor precipitating rapid capacity decay, especially under a high cutoff voltage and long-time cycling [37]. Figure 5c and d presents HR-TEM images of the CEI covering the cycled NCM622 cathodes. A uniform and thin CEI layer (∼6.59 nm) is observed on the cathode cycled in PTIDOL, indicating outstanding compatibility between the Ni-rich cathode and PTIDOL. Conversely, a thicker and uneven CEI layer (∼13.7 nm) is observed in PTEDOL. The augmented thickness of the CEI implies more severe parasitic reactions between the electrolyte and high-voltage cathode, leading to rapid capacity decay of the Li|PTEDOL|NCM622 cell [38].

As shown in Fig. 5e–h, further identification of the CEI composition on the cathode after cycling at 4.5 V is conducted using X-ray photoelectron spectroscopy (XPS) with Ar^+^ depth analysis. The CEI of the NCM622 cathode in the Li|PTEDOL|NCM622 cell exhibits higher signal intensities of C−O, O−C=O and Li_2_CO_3_ (attributed to the decomposition of the polymer network [39]) and lower signal intensities of LiF and B-F. This indicates that PTEDOL undergoes more severe oxidative decomposition at high voltage, forming a CEI primarily composed of organic compounds. By comparison, the CEI on the NCM622 cathode in the Li|PTIDOL|NCM622 cell displays a more pronounced peak of LiF (685.0 eV) in the F 1s spectra. After sputtering, the signal intensity of LiF and B-F in PTIDOL is enhanced, indicating that CEI is rich in inorganic components derived from TFSI^−^ and DFOB^−^. The notable increase in LiF content is essential for significantly improving the oxidative stability of PTIDOL. This is because LiF offers exceptional physical and chemical stability while exhibiting low electronic conductivity, making it an advantageous choice as an interfacial component across a range of battery systems [40]. The results demonstrate that PTIDOL undergoes less decomposition on the high-voltage cathode side due to its high oxidative stability, which is conducive to maintaining the structural integrity of the cathode particles.

XRD measurements are used to study the phase reconstruction of the NCM622 cathodes with different electrolytes (Fig. S24). Compared to pristine NCM622, the (003) peak in the XRD pattern of NCM622 cycled in PTEDOL shifts significantly downward, suggesting severe irreversible phase reorganization in the cathode. In contrast, the (003) peak in the XRD patterns for NCM622 cycled in the PTIDOL exhibits a slighter leftward shift compared to pristine NCM622. These results indicate the beneficial influence of PTIDOL in preserving the structural stability of NCM622 when compared to PTEDOL [41].

### Effect on Li metal electrode

**Figure 6.**
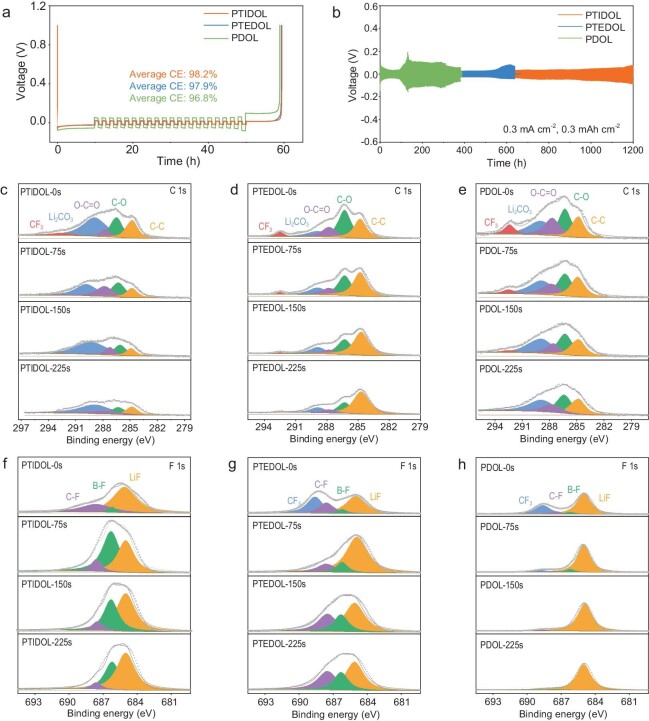
Li metal compatibility and characterization of the SEI on Li metal. (a) Coulombic efficiency plots of the Li||Cu half-cell with PTIDOL, PTEDOL and PDOL. (b) Galvanostatic cycling curves of the Li||Li symmetric cells with PTIDOL, PTEDOL and PDOL at 0.3 mA cm^−2^ and 0.3 mAh cm^−2^. (c–h) XPS depth profiles of (c–e) C 1s and (f–h) F 1s of lithium metal harvested from the Li||Li symmetric cell at a current density of 0.3 mA cm^−2^ with a capacity of 0.3 mA h cm^−2^ after 50 cycles.

To evaluate the cycling reversibility of Li plating/stripping on the side of the Li metal anode, Coulombic efficiency (CE) tests are conducted using the Aurbach CE tests at 0.5 mA cm^−2^ (Fig. 6a). The results indicate that the average CE within 20 cycles of the Li|PTIDOL|Cu half-cell is 98.2%, which is higher than that of the Li|PTEDOL|Cu and Li|PDOL|Cu cells. The higher CE implies that PTIDOL has satisfactory compatibility with the lithium metal anode. Considering the above results, which promote the cycle stability of Li metal, the cycle test of symmetric cells is conducted to further elucidate the advantages of PTIDOL for the Li metal anode in cycling, as displayed in Fig. S25. At a current density of 0.1 mA cm^−2^ (0.1mAh cm^−2^), the Li|PTIDOL|Li symmetric cell stably cycles for 3200 h. Furthermore, a Li||Li symmetric cell assembled with PTIDOL, PTEDOL and PDOL is tested at a higher current density 0.3 mA cm^−2^ with a fixed capacity of 0.3 mAh cm^−2^ (Fig. 6b). The short circuit of Li|PTEDOL|Li and Li|PDOL|Li symmetric cells occurs after 600 hours and 380 hours of cycling respectively. In contrast, the Li|PTIDOL|Li symmetric cell demonstrates a superior cycle life of 1200 hours, which reveals the formation of a more stable solid electrolyte interphase (SEI) and subsequent stable reversibility of Li plating/stripping. Furthermore, the Li|PTIDOL|Li symmetric cell maintains a consistently low and stable interfacial resistance during cycling, whereas the interfacial resistance of Li|PTEDOL|Li and Li|PDOL|Li symmetric cells gradually increases (Fig. S26). The Li|PTIDOL|Li symmetric cell also shows stable electrochemical cycling performance at a current density of 0.5 mA cm^−2^ for 300 h (Fig. S27). The better cycling reversibility of PTIDOL in Li||Cu and Li||Li cells regarding plating/stripping can mainly be attributed to: (i) the elimination of −OH groups, which enhances the stability of the Li metal anode [19]; (ii) the increase in t_Li+_, which is advantageous for reducing concentration polarization, promoting uniform lithium deposition and suppressing the growth of lithium dendrites [42]. Surface morphology and cross-sectional views of the Li metal anode after 100 hours of cycling are investigated by SEM (Fig. S28). The Li metal surface after cycling in PDOL exhibits a porous and rough surface morphology, which increases interfacial resistance and leads to rapid cell failure during repeated plating/stripping cycles. The Li metal in PTEDOL exhibits a relatively dense but still rough surface morphology, a result of the avoidance of parasitic reactions between the highly reactive terminal −OH groups and the strongly reducing Li metal. In contrast, the Li metal surface in PTIDOL appears smooth and uniform, indicating a uniformly dense Li deposition in PTIDOL. The high current density tolerance of polyether electrolytes is evaluated by cycling Li||Li symmetric cells at different current densities. As shown in Fig. S29, the Li|PTEDOL|Li and Li|PDOL|Li symmetric cell suffers a sharp increase in overpotential and subsequent failed at current densities of 1.1 mA cm^−2^ and 0.5 mA cm^−2^, respectively. By comparison, the Li|PTEDOL|Li symmetric cell maintains relatively stable voltage polarization even at 1.4 mA cm^−2^, indicating that PTIDOL exhibits better tolerance to high current densities compared to PTEDOL and PDOL due to the higher ionic conductivity and improved interfacial stability.

The structure of the SEI is systematically investigated by XPS depth profiling (Fig. 6c–h). In the C 1s spectra, compared to PDOL, PTIDOL and PTEDOL exhibit lower signal intensities at different sputtering times, which originate from side reactions within the polymer network. Due to the elimination of highly reactive terminal −OH groups, parasitic reactions between the strongly reducing Li metal and the electrolyte are inhibited. In the F 1s spectra, PTIDOL demonstrates a higher signal intensity of LiF compared to the other two electrolytes. The high mechanical modulus and surface energy of LiF are crucial for the formation of a stable and uniform SEI, which is essential for suppressing lithium dendrite growth [43,44]. More importantly, in the N1s spectrum (Fig. S30), PTIDOL exhibits a higher signal intensity of Li_3_N, and the Li_3_N signal intensity remains almost constant with increasing sputtering depth, indicating a uniform distribution of Li_3_N throughout the SEI. The lowest unoccupied molecular orbital (LUMO) of PTIDOL is primarily located at the center of TGIC (−3.65 eV), which is lower than that of PDOL (1.04 eV) (Fig. S31). The lower LUMO of TGIC facilitates preferential reduction on the Li metal anode, thereby inducing the formation of a Li_3_N-rich SEI on the lithium metal anode. Li_3_N has advantages such as a low Li^+^ diffusion barrier, high ionic conductivity and low electronic conductivity [45], which facilitate steady Li deposition/stripping during long-term cycling. Overall, the Li_3_N-LiF composite SEI provides high mechanical strength and fast interfacial kinetics to buffer significant volumetric changes during Li deposition and to achieve even Li deposition [46].

## CONCLUSIONS

In this work, we propose a local charge homogenization strategy to reinforce the high-voltage tolerance of polyether electrolytes, aimed at addressing the challenges associated with the compatibility of conventional polyether electrolytes and high-voltage cathodes. Comprehensive simulations and characterizations reveal the critical role of TGIC as a functional crosslinker in PTIDOL, in which the unique structure triggers Li^+^ solvation structure reorganization to induce a uniform charge distribution at EO sites, thereby improving the oxidative stability of the polyether main chain. Moreover, this strategy can address the oxidation issue of the terminal units by eliminating the terminal −OH groups. Due to the enhanced oxidative stability of both the host and terminal, the decomposition of PTIDOL under high voltage is effectively inhibited, thus facilitating the formation of a thin and uniform CEI. Consequently, the Li|PTIDOL|NCM622 cell exhibits an impressive 400-cycle lifespan at an ultra-high cutoff voltage of 4.7 V (81.8% capacity retention), the best reported result for polyether electrolytes to date. Moreover, PTIDOL demonstrates the ability to cycle 150 times (with an 89.4% capacity retention) in a 2-Ah pouch cell with low N/P ratios (1.13) and high-loading cathodes (20.3 mg cm^−2^). This work presents a new approach with regard to the design of high-voltage-tolerant polyether electrolytes and their industrial application.

## METHODS

Detailed preparation and characterization methods for materials are available in the online supplementary data.

## Supplementary Material

nwae436_Supplemental_File
