# Supplementary material for: Local charge homogenization strategy enables ultra-high voltage tolerance of polyether electrolytes for 4.7 V lithium metal batteries
Source: Natl Sci Rev. 2024 Dec 3;12(2):nwae436. doi: 10.1093/nsr/nwae436 (PMC11737385; doi:10.1093/nsr/nwae436)
Supplement: nwae436_Supplemental_File [file nwae436_supplemental_file.pdf]

Supporting Information

Local charge homogenization strategy enables ultra-high voltage tolerance of polyether electrolytes for 4.7V lithium metal batteries

Yuanlong Wu[1,†], Piao Luo[1,†], Kexin Su[1,†], Mao Yu[2], Xin Song[3], Lianzhan Huang[1], Shaocong Zhang[4], Huiyu Song[1], Li Du[1], Weishu Liu[2,*] and Zhiming Cui[1,*]

[1]Guangdong Provincial Key Laboratory of Fuel Cell Technology, School of Chemistry and Chemical Engineering, South China University of Technology, Guangzhou 510641, China.

[2]Department of Materials Science and Engineering, Southern University of Science and Technology, Shenzhen 518055, China

[3]Guangdong Provincial Key Laboratory of Advanced Energy Storage Materials, School of Materials Science and Engineering, South China University of Technology, Guangzhou 510641, China.

[4]School of Software Engineering, South China University of Technology, Guangzhou 510006, China

***Corresponding authors.** E-mails: zmcui@scut.edu.cn; liuws@sustech.edu.cn

† Equally contributed to this work.

Table of contents

Section S1. Materials and Methods

Section S2. Characterizations

Section S3. Electrochemical Measurements and Computational Methods

Section S4. Supplementary Figures and Tables

Section S5. Supplementary References

Section S1. Materials and Methods

Materials: Triglycidyl Isocyanurate (TGIC), trimethylolpropane triglycidyl ether(TTE) are purchased from Aladdin. 1,3-dioxolane (DOL), fluoroethylene carbonate (FEC), lithium difluoro(oxalate)borate (LiDFOB), and lithium bis(trifluoromethanesulfonyl)imide (LiTFSI) are purchased from Duoduo chemical company in Suzhou.

Electrolyte Preparation: PTIDOL is prepared by in-situ polymerization of precursor solution composed of crosslinker (TGIC), monomer (DOL), solvent (FEC), and two lithium salts (LiDFOB and LiTFSI). For the preparation of PTIDOL, first, TGIC is added into the mixed FEC/DOL (1:3 by volume), with the weight ratio of m(TGIC):m(DOL) = 1:20. Second, 0.6 mol L^{-1} LiDFOB and 0.6 mol L^{-1} LiTFSI are dissolved in the mixture of TGIC, DOL and FEC, and mix thoroughly to obtain the precursor solution. The precursor solution is injected into the polyethylene (PE) separator (25 μm thickness), and the cell is assembled and sealed. The precursor solution is heated at 60 ℃ to form the PTIDOL. PTEDOL and PDOL are prepared in the same process, with the difference being that the precursor solution of PTEDOL contains the crosslinker TTE, while PDOL does not contain any crosslinker. All processes of electrolyte preparation are conducted in an Ar-filled glove box (Mikrouna, $[O_2]$ < 0.01 ppm, $[H_2O]$ < 0.01 ppm).

Electrode Preparation: $LiNi_{0.6}Co_{0.2}Mn_{0.2}O_2$(NCM622) and $LiNi_{0.8}Co_{0.1}Mn_{0.1}O_2$ (NCM811) cathodes are prepared by casting a uniformly dispersed slurry of active materials, super P, and poly(vinylidene fluoride) (PVDF) (8:1:1) onto Al foil and dry for 12 h in drying oven at 80 ℃. The cathodes have an active material mass loading of about 2 mg cm^{-2} . Li foil (500 μm) is purchased from China Energy Lithium Co., Ltd.

Cell Assembly: To evaluate the ionic conductivity of PTIDOL, PTEDOL and PDOL, the electrolyte is sandwiched between two pieces of stainless steel (SS), respectively.

As for linear sweep voltammetry (LSV), SS|SPEs|Li foil batteries are prepared for the test. To assemble the Li|SPEs|Li symmetric cell, the electrolyte is sandwiched with two Li foils (d =10 mm) in coin cells. Coin cells or pouch cells are assembled with the above cathodes, Li anode, and PE separator in the Ar-filled glove box. All coin cells assembled are conducted in an argon-filled glove box (H_2O < 0.01 ppm, O_2 < 0.01 ppm). After the injection of the precursor solution, the cells are heated at 60 ℃ for 12 h.

Section S2. Characterizations

The Carl Zeiss SEM is utilized to observe the morphologies of electrolytes and electrodes. The transmission electron microscopy images are characterized by a FEI Tecnai G2 F20 transmission electron microscope. Fourier transform infrared (FTIR) spectra using Nicolet-IS20 (Thermo Fisher Scientific) are utilized to investigate the structure of the electrolytes. The XRD patterns of the samples are obtained from a MiniFlex (Rigaku, Japan) with Cu Kα radiation. Raman spectroscopic measurement is performed by WiTec alpha300R confocal Raman microscope. X-ray photoelectron spectroscopy (XPS, K-Alpha) etching depth is employed to explore the surface and inner compounds on the cycled electrodes using Al Kα radiation. The glass transition temperature of electrolyte is investigated by differential scanning calorimetry (DSC, Netzsch DSC 200 F3, heating rate: 10 K min^{-1}). ^{7}Li and ^{13}C solid-state nuclear magnetic resonance (NMR) spectroscopy is acquired on Agilent 600M. The NCM622 cathode for XRD, SEM, TEM, and XPS characterization was obtained by disassembling the cell. The sample was then washed with DOL more than five times to remove residual electrolytes before characterization.

Section S3. Electrochemical Measurements, Calculation, and Computational Methods

Electrochemical Measurements: PTIDOL, PTEDOL and PDOL used for electrochemical measurements are in-situ fabricated in the corresponding cells. The electrochemical tests of Li||Li, Li||Cu, Li||NCM811, and Li||NCM622 are performed

using a LAND battery testing system (Land CT3002A model, Wuhan LAND Electronics.Ltd.).

Electrochemical impedance spectroscopy (EIS) tests are conducted at the Autolab Electrochemical Instrumentation (Metrohm) in a frequency range from 0.1 Hz to 100 kHz with a perturbation amplitude of 10 mV. The testing temperatures are ranged from 25 to 65 ℃ and the ionic conductivity σ is calculated by the following equation:

$$\sigma = \frac{L}{SR}$$

where L represents the thickness (cm) of the electrolyte membrane, R represents the bulk resistance (Ω), and S corresponds to the contact area (cm^2) between SS and electrolyte.

The activation energy of Li^+ transport is calculated based on equation :

$$\sigma = A_{exp}\ (-\frac{E_a}{RT})$$

where A is the pre-exponential factor, Ea is the activation energy of Li^+ transport, R is the ideal gas constant, and T is the testing absolute temperature.

LSV measurements are conducted at a sweep rate of 1 mV s^{-1} from 3 to 6 V.

The Li-ion transfer number (tLi^+) is calculated by the following equation:

$$t_{Li^+} = \frac{I_s(\Delta V - I_0 R_0)}{I_0(\Delta V - I_s R_s)}$$

where I_o and I_s are the initial and steady currents during polarization, R_o and R_s are the resistance values before and after the polarization, and ΔV (10 mV) is the constant applied voltage during polarization.

The electronic conductivities were tested in symmetric cells with stainless-steel spaces as electrodes and conducted via Chronoamperometry (CA) tests with an applied voltage of 500mV.

Computational Methods:

Density functional theory (DFT) calculations, which achieve the Becke atomic charge with atomic dipole moment correction, the electrostatic potentials (ESPs), binding energies and HOMO energy level, are conducted by Gaussian (G16) program with Becke's three-parameter hybrid method using the Lee-Yang-Parr correlation functional (B3LYP) at 6-311++G(d,p) level. The universal solvation model of SMD is employed to consider the solvation effect. Structural optimization and frequency analysis are both conducted at this same theoretical level. All optimized structures meet the default convergence criteria and showed no imaginary frequencies. Becke atomic charge with atomic dipole moment correction is conducted using Multiwfn[1], while HOMO energy level diagrams are illustrated using VMD. The oxidation potential E_{ox} vs. Li/Li^+ is calculated as follows:

$$E_{OX} = \frac{G(M^+) - G(M)}{F} - 1.4V$$

where G(M) and $G(M^+)$ are the free energy of species M, its oxidized forms at 298.15 K, respectively. F is the Faraday constant.

The formula for the binding energy (E_{BE}) is as follows:

$E_{BE} = Etotal - \sum Efrag$

Where Etotal represents the overall electronic energy of the complexes, Efrag denotes the electronic energies of individual fragments.

The classical MD simulation is performed using the Lammps software package from Sandia National Laboratory to investigate the solvation structure of electrolytes. Three simulated systems are created, one for each electrolyte under study, PTIDOL, PTEDOL and PDOL. PTIDOL(or PTEDOL PDOL), FEC, LiTFSI, and LiDFOB are added to an 10 ×10 ×10 nm simulation box, in accordance with the stoichiometry. The force-field parameters of electrolytes are assessed with General Amber force field (GAFF) parameters[2]. For all molecule, density functional theory calculation is performed at the B3LYP/6-311G(d, p) level with Gaussian 16 package, and the resulting molecular structure is analyzed by the Multiwfn program to derive the RESP

charges. The non-bonded interactions between any atomic pair are calculated by the Lorentz–Berthelot combination rules. Given that all the atoms in the system are carrying a charge, the electrostatic interaction is described by the Coulomb potential with the inner and outer cutoff radius of 10.0 and 12.0 Å, respectively. The long-range Coulombic interactions are solved using the Particle–Particle Particle–Mesh algorithm with an accuracy value of 10^{-5} .

The simulation system is first optimized to the local minimum of energy by the conjugate gradient method. Then, Under high pressure and temperature, a set of NVT/NPT processes are conducted to equilibrate systems. The processes broadly based on previous works[3] (details in the Table S1). Finally, the MD simulation is carried out under the NVT ensemble maintaining at a temperature of 303 K with Nosé-Hoover thermostat. The time step is set to 1.0 fs and the total simulation time is 10.0 ns.

Table S1. The equilibration routine for simulated systems

Stage	Stage duration [ns]	Total time [ns]	Temperature [K]	Pressure [MPa]
Energy minimisation	-	-	-	-
NPT	0.10	0.10	353	0-100
Energy minimisation	-	-	-	-
NPT	0.50	0.60	353	0-400
NPT	0.40	1.00	353	400
NPT	0.60	1.60	353	400-0.1
NPT	0.40	2.00	353-453	0.1
NPT	0.40	2.40	453-353	0.1
NPT	0.30	2.70	353	0.1-400
NPT	0.30	3.00	353	400-0.1
NVT	0.80	3.80	353	-
NPT	1.20	5.00	353	0.1

Section S4. Supplementary Figures and Tables

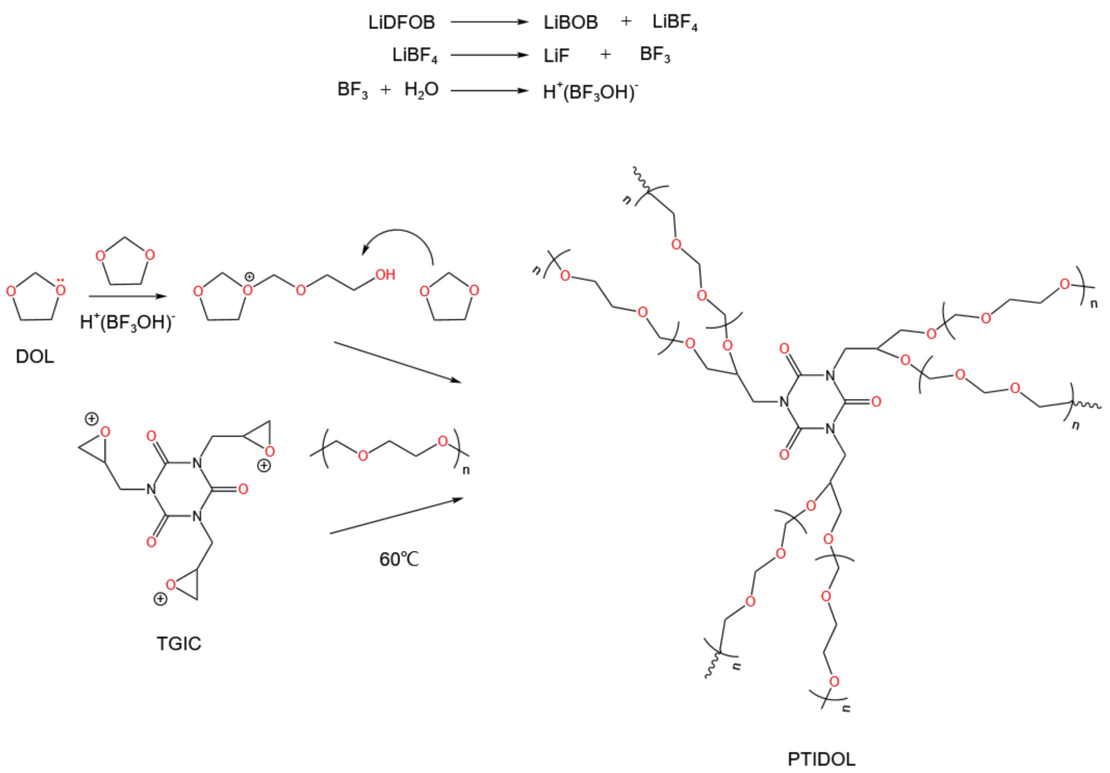

Figure S1. Schematic diagram showing the polymerization process of PTIDOL.

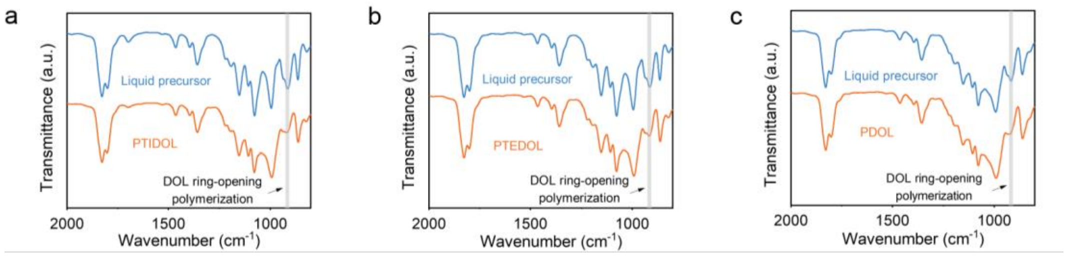

Figure S2. FTIR spectrum of PTIDOL, PTEDOL, PDOL, and liquid precursor from 800 cm^{-1} to 2000 cm^{-1}.

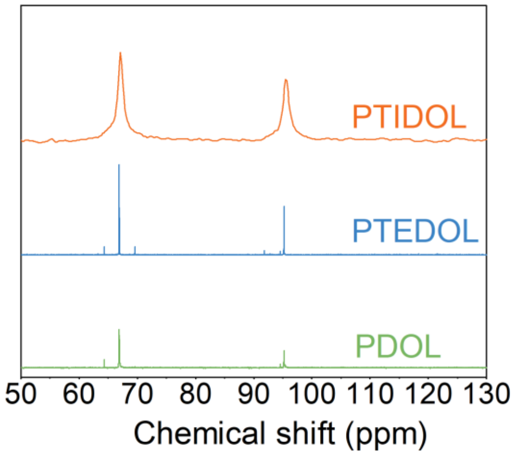

Figure S3. ^{13}C NMR spectra of PTIDOL, PTEDOL, and PDOL.

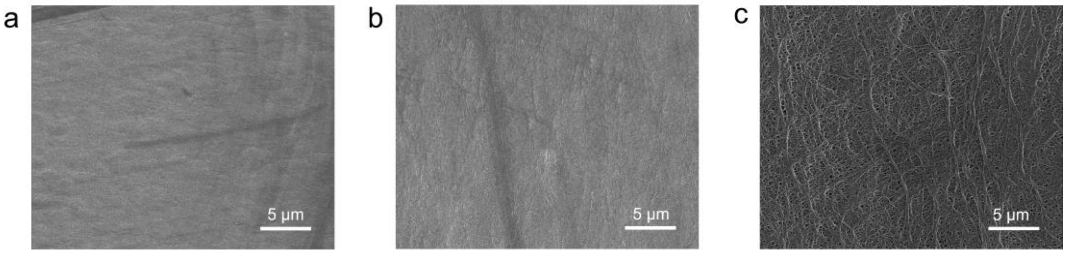

Figure S4. Scanning electron microscopy (SEM) images of (a) PTEDOL, (b) PDOL and (c) PE membrane.

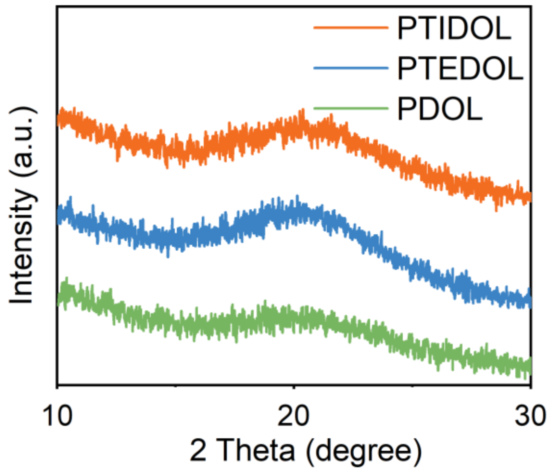

Figure S5. XRD patterns of PTIDOL, PTEDOL, and PDOL samples.

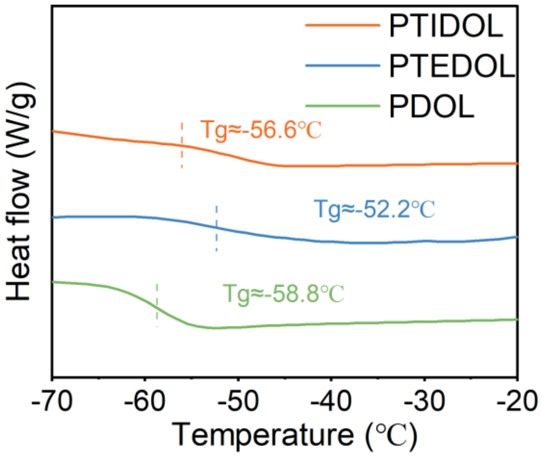

Figure S6. DSC curves of PTIDOL, PTEDOL, and PDOL

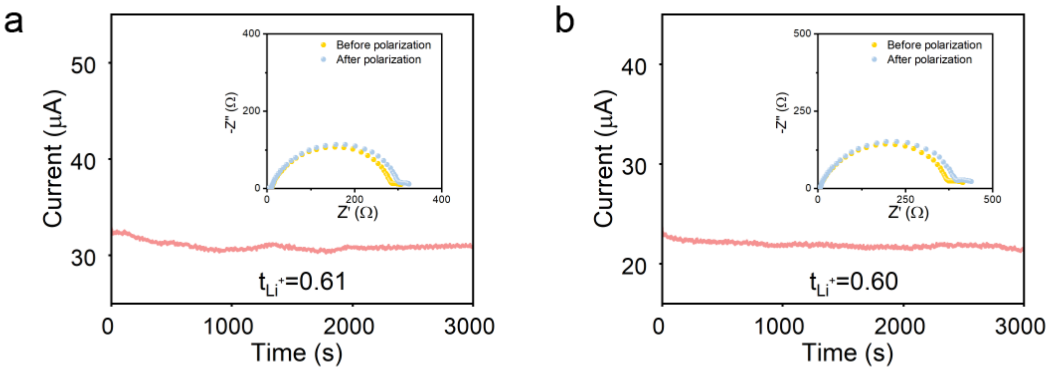

Figure S7. Chronoamperometry polarization curves and the impedance spectra before and after polarization of Li||Li symmetric cells with (a) PTEDOL and (b) PDOL.

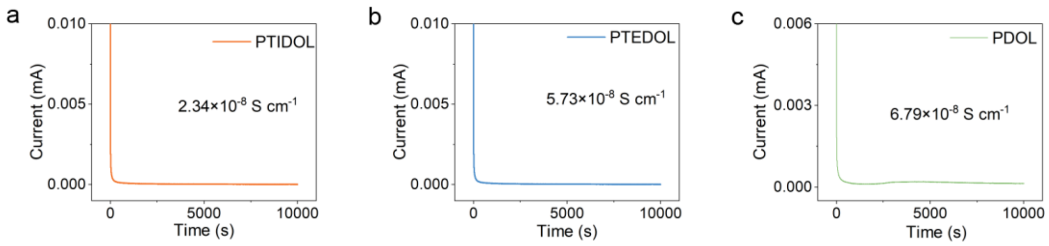

Figure S8. Electronic conductivities tests of (a) PTIDOL, (b) PTEDOL, and (c) PDOL.

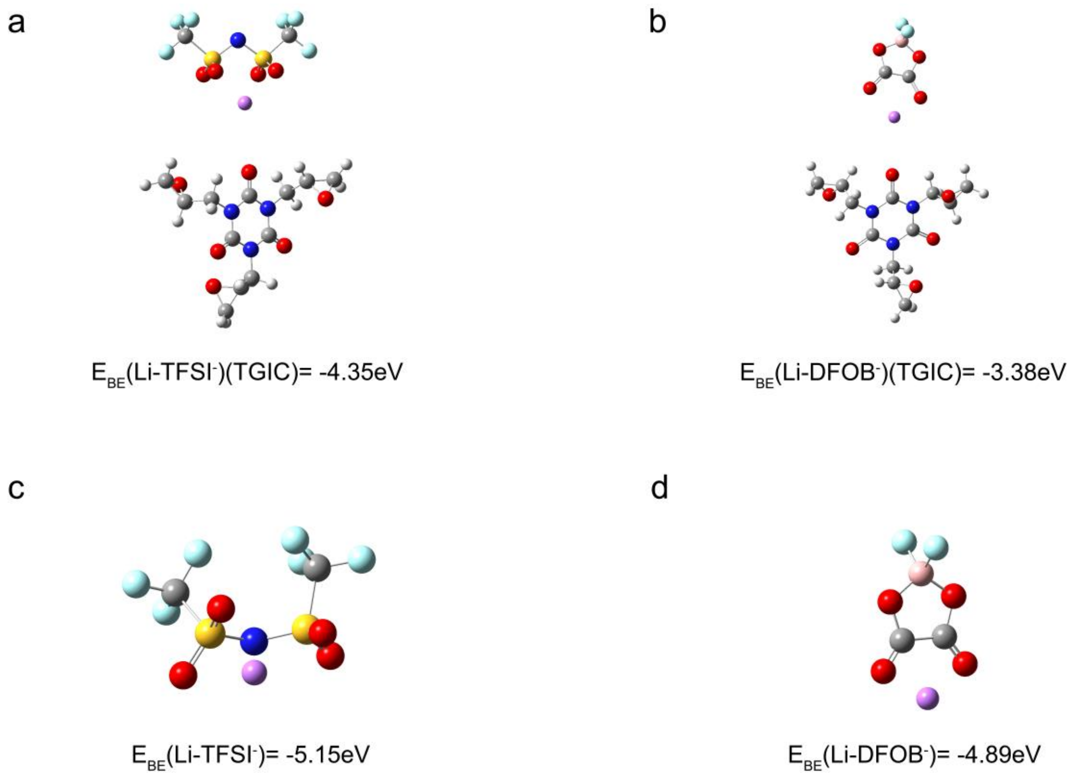

Figure S9. The binding energy of Li^{+}/anions (a-b)within TGIC (c-d)without TGIC

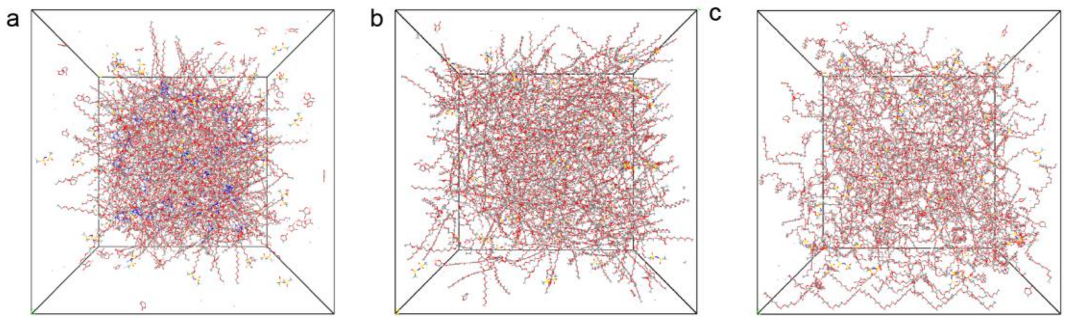

Figure S10. The initial MD models of (a) PTIDOL, (b) PTEDOL, and (c) PDOL.

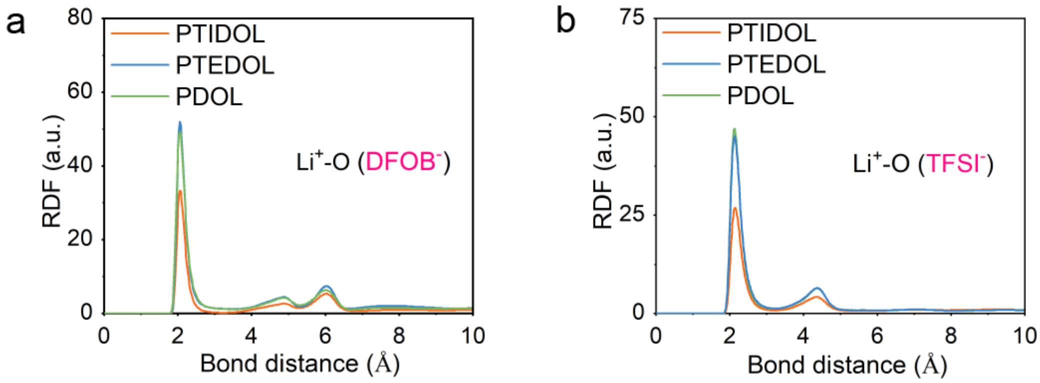

Figure S11. Calculated radial distribution functions of (a) Li^+-O ($DFOB^-$) and (b) Li^+-O ($TFSI^-$) for PTIDOL, PTEDOL, and PDOL.

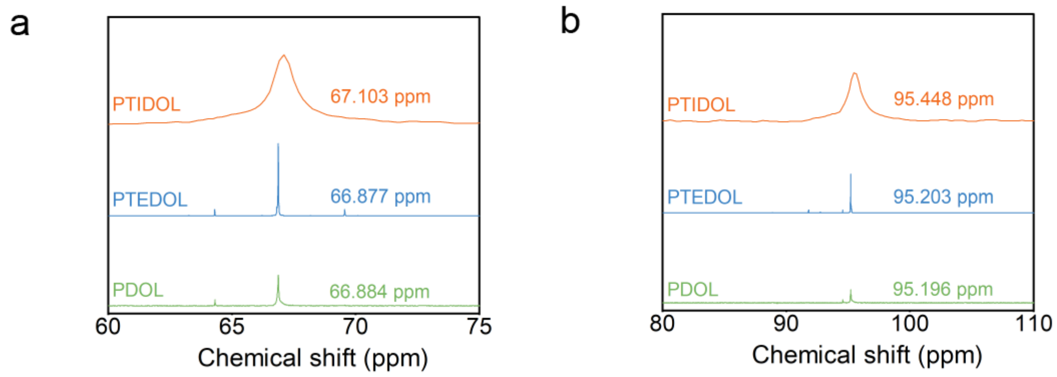

Figure S12. ^{13}C NMR spectra of PTIDOL, PTEDOL and PDOL from (a) 60ppm to 75ppm and (b) 80ppm to 110ppm.

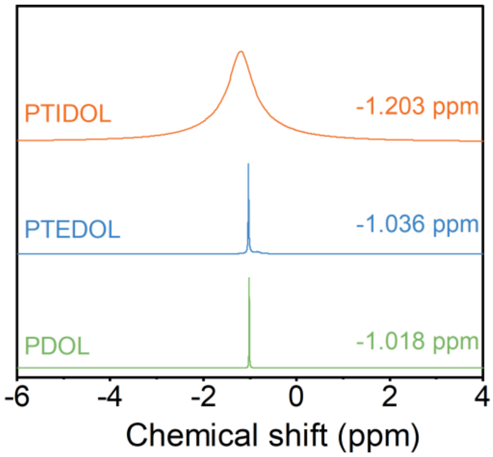

Figure S13. ^{7}Li NMR spectra of PTIDOL, PTEDOL, and PDOL.

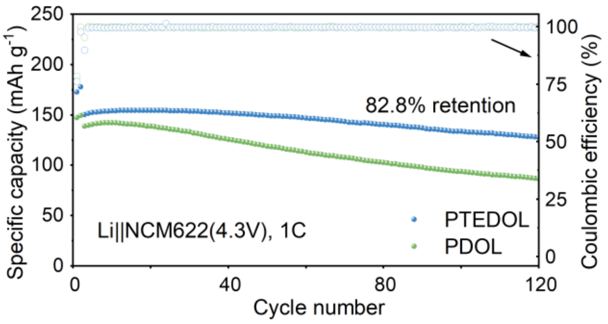

Figure S14. Cycling performances of Li||NCM622 cells using PTEDOL and PDOL at 1 C at charge cut-off voltages of 4.3 V.

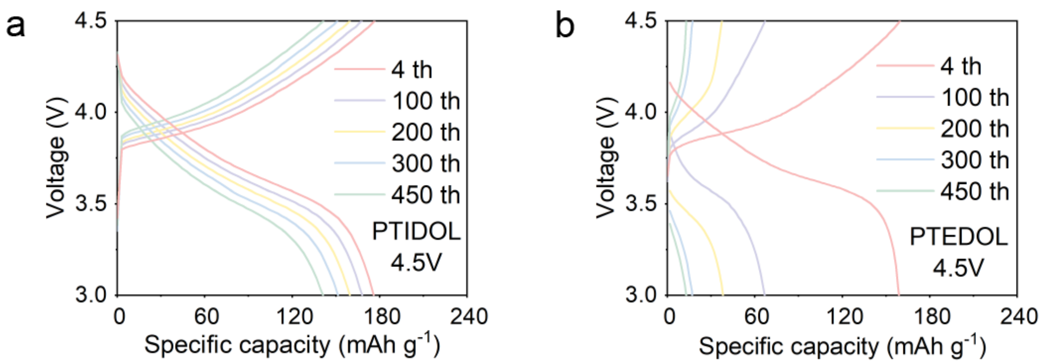

Figure S15. Corresponding voltages profiles of Li||NCM622 cells using (a) PTIDOL and (b) PTEDOL at 1 C at charge cut-off voltage of 4.5 V.

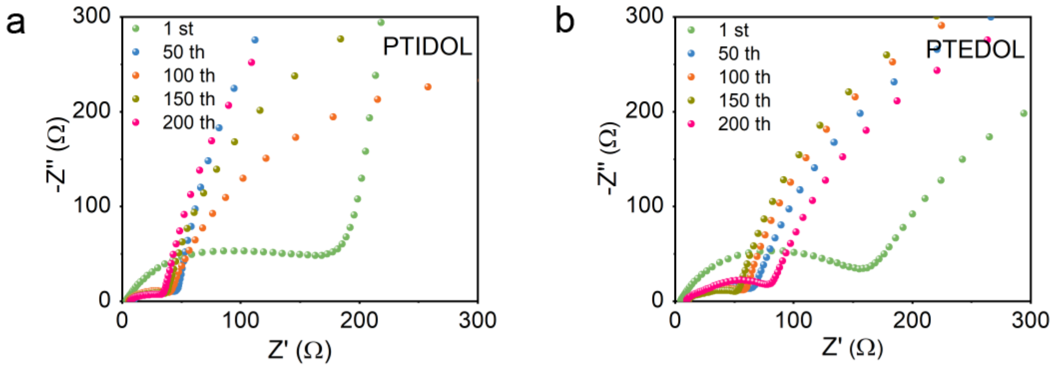

Figure S16. EIS spectra of Li||NCM622 cells using (a) PTIDOL and (b) PTEDOL electrolytes after different cycles at 1 C at charge cut-off voltage of 4.5 V.

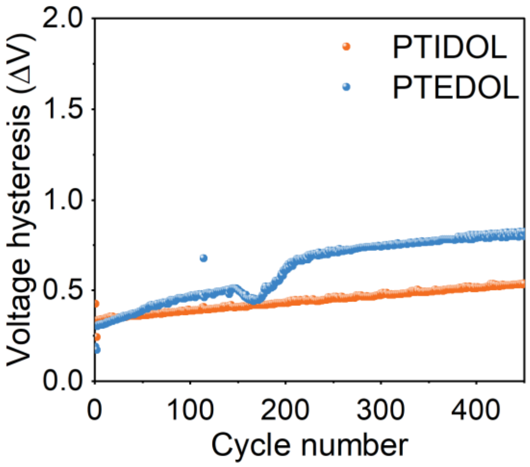

Figure S17. Hysteresis voltage of Li||NMC622 cell at 1C at charge cut-off voltage of 4.5 V.

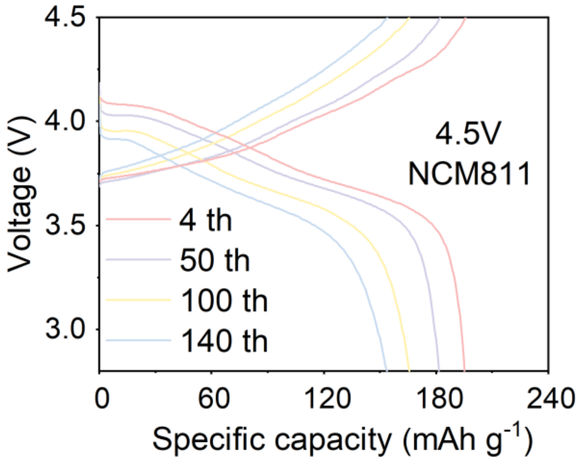

Figure S18. Corresponding voltages profiles of Li||NCM811 cells using PTIDOL at 1 C at charge cut-off voltage of 4.5 V.

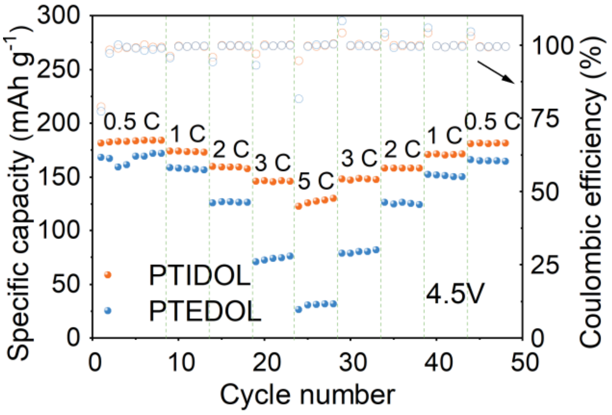

Figure S19. Rate performance of Li||NCM622 cell at charge cut-off voltage of 4.5 V.

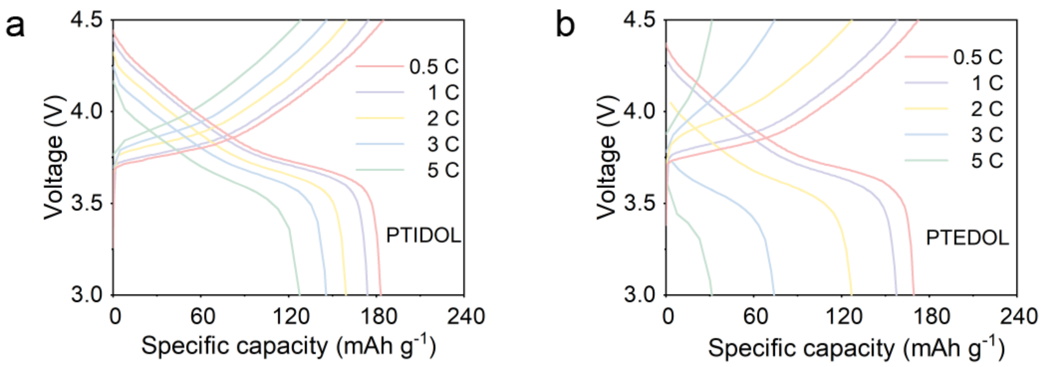

Figure S20. Corresponding voltages profiles of Li||NCM622 cells using (a) PTIDOL and (b) PTEDOL at 0.5 C, 1 C, 2 C, 3 C, and 5 C at charge cut-off voltage of 4.5 V.

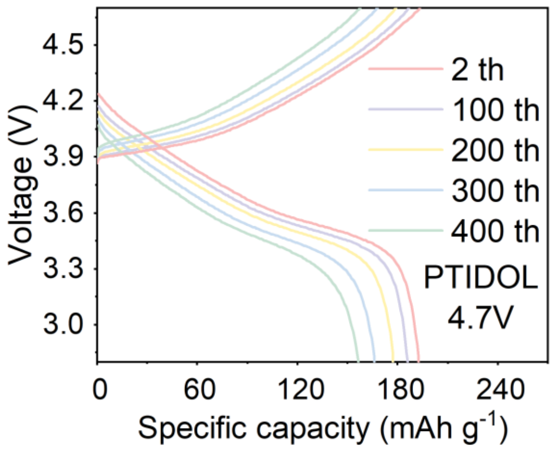

Figure S21. Corresponding voltages profiles of Li||NCM622 cells using PTIDOL at 1 C at charge cut-off voltage of 4.7 V.

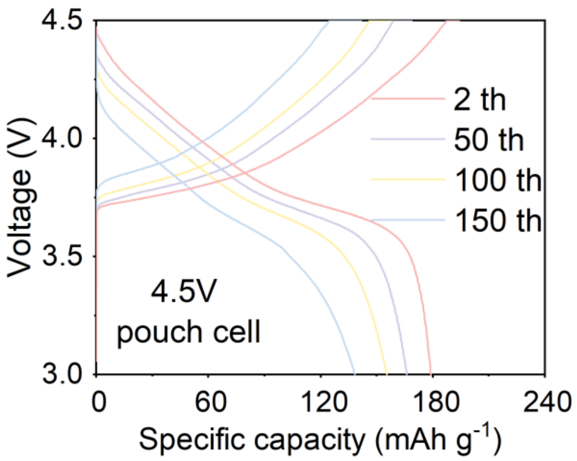

Figure S22. Corresponding voltages profiles of Li||NCM622 pouch cells using PTIDOL at 0.5 C at charge cut-off voltage of 4.5 V.

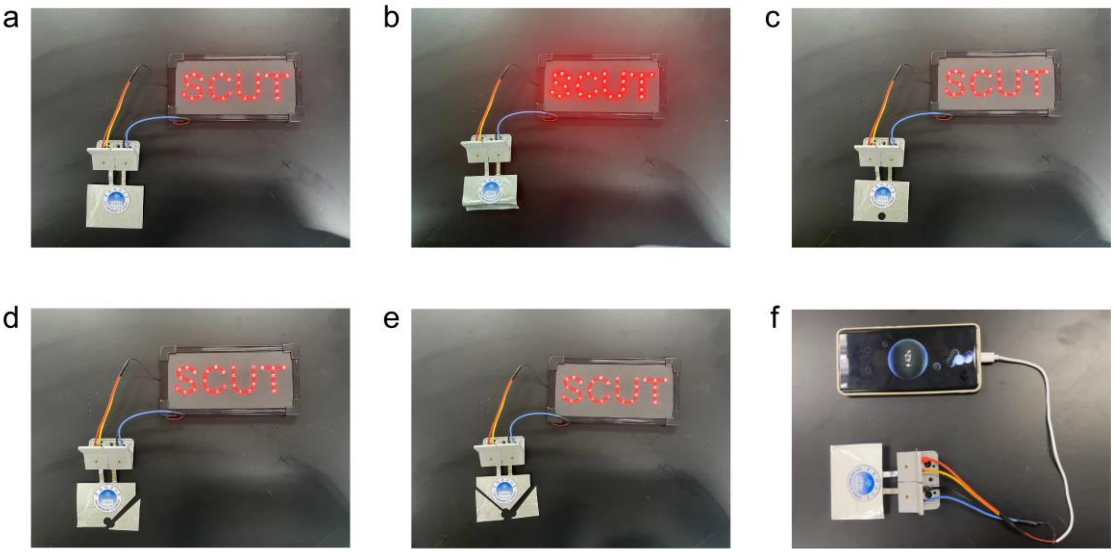

Figure S23. (a-e) Safety test of Li|PTIDOL|NCM622 pouch cell under various conditions including bending, cutting, and punching. (f) Li|PTIDOL|NCM622 pouch cell to charge the phone.

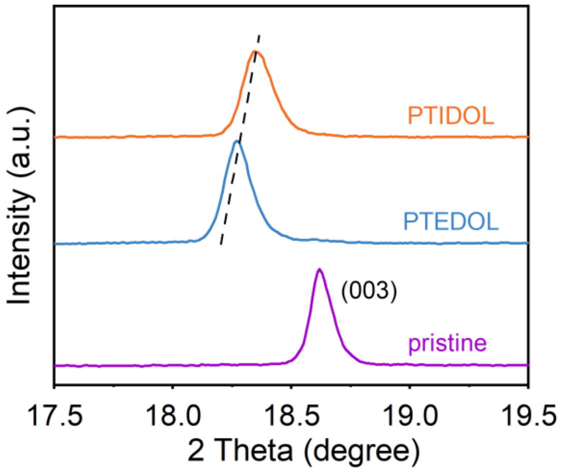

Figure S24. XRD patterns of NCM622 cathode after 50 cycles.

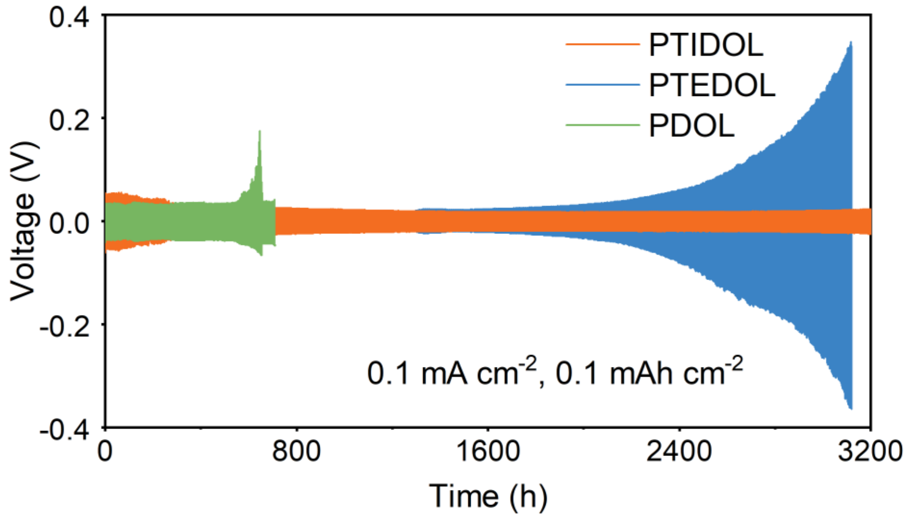

Figure S25. Galvanostatic cycling curves of Li||Li symmetric cell with PTIDOL, PTEDOL, and PDOL at 0.1 mA cm^{-2} and 0.1 mAh cm^{-2}.

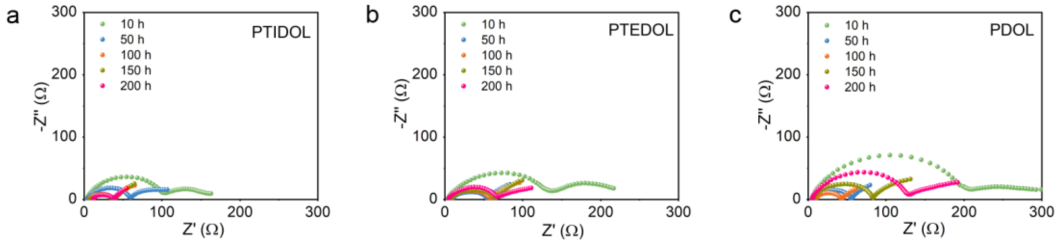

Figure S26. EIS spectra of the Li||Li symmetric cells using (a) PTIDOL, (b) PTEDOL, and (c) PDOL after different plating/stripping cycles at a current density of 0.3 mA cm^{-2} with a capacity of 0.3 mAh cm^{-2}.

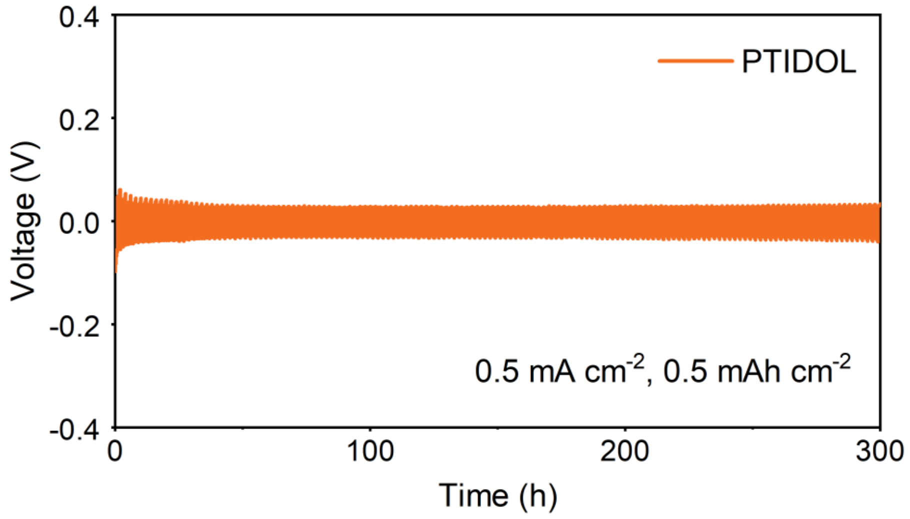

Figure S27. Galvanostatic cycling curves of Li||Li symmetric cell with PTIDOL at 0.5 mA cm^{-2} and 0.5 mAh cm^{-2}.

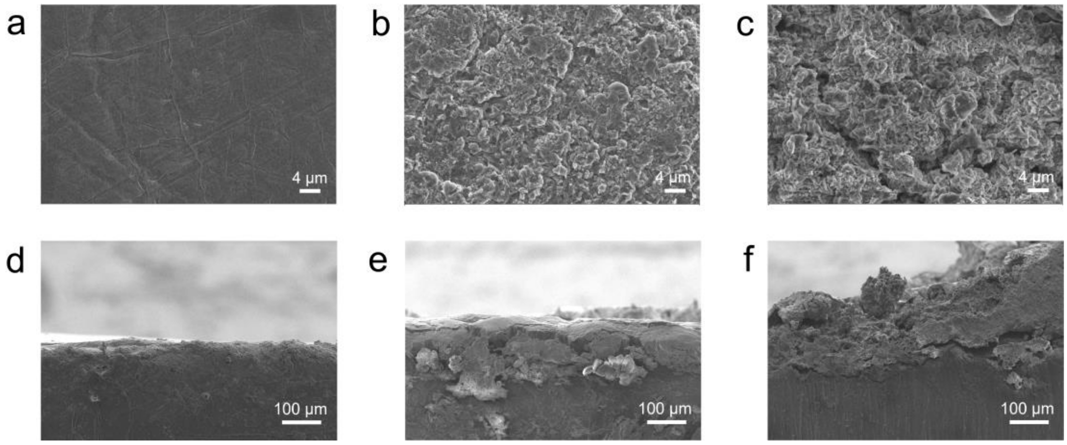

Figure S28. SEM images illustrating surface morphologies and cross-section views of the Li-metal anodes with (a,d) PTIDOL , (b,e) PTEDOLand (c,f) PDOL in Li||Li symmetric cells at 0.3 mA cm^{-2} and 0.3 mAh cm^{-2} after 50 cycles.

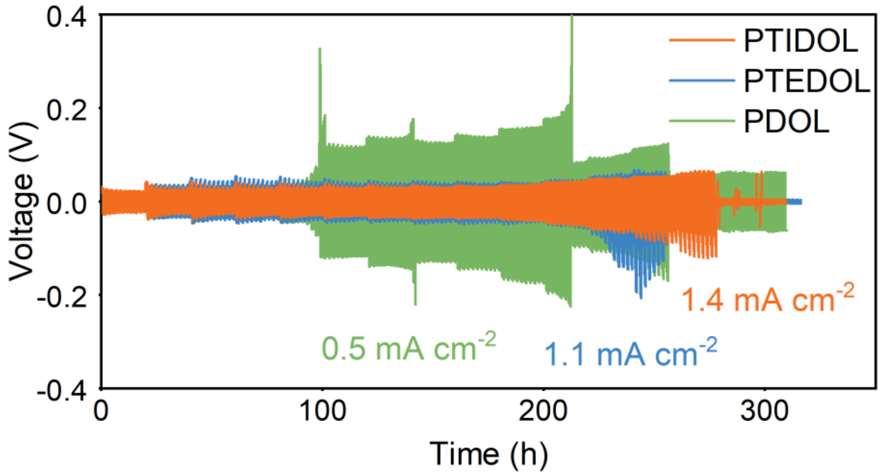

Figure S29. Critical current density tests of Li||Li symmetric cell using PTIDOL, PTEDOL, and PDOL.

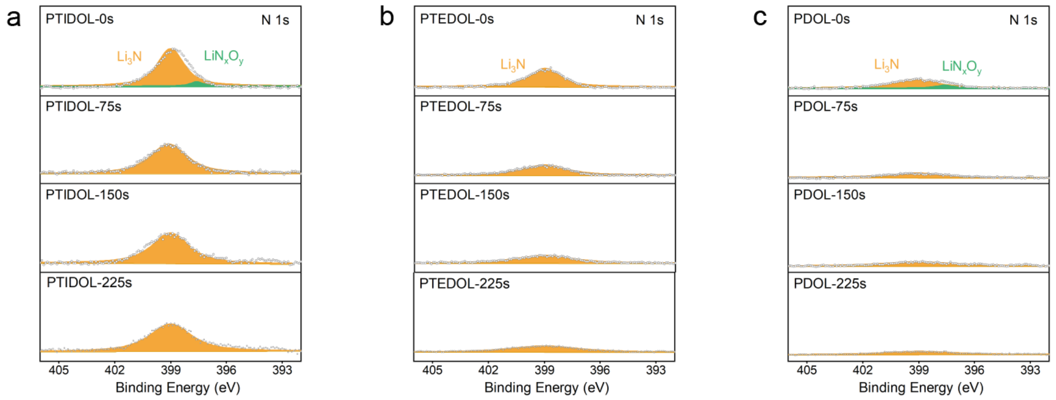

Figure S30. XPS depth profiles of N 1s of lithium metal harvested from Li||Li symmetric cell at a current density of 0.3mA cm^{-2} with a capacity of 0.3 mA h cm^{-2} after 50 cycles.

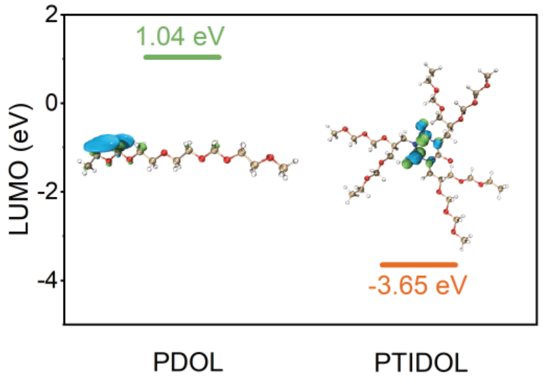

Figure S31. Calculated LUMO energy values of PTIDOL and PDOL.

Table S2. Becke atomic charge of Li^{+}-2EO

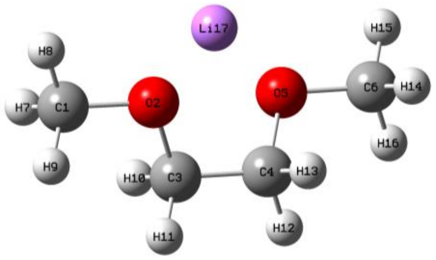

Atom	charge
C1	-0.007
O2	-0.306
C3	0.128
C4	0.102
O5	-0.259
C6	-0.029
H7	0.063
H8	0.077
H9	0.070
H10	0.087
H11	0.036
H12	0.047
H13	0.088
H14	0.054
H15	0.078
H16	0.054
Li17	-0.286

Table S3. Becke atomic charge of Li^+-5EO

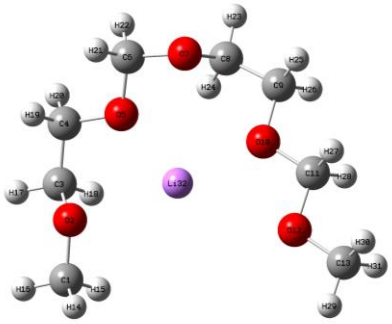

Atom	charge
C1	-0.042
O2	-0.449
C3	0.076
C4	0.117
O5	-0.612
C6	0.284
O7	-0.386
C8	0.058
C9	0.025
O10	-0.352
C11	0.201
O12	-0.392
C13	-0.037
H14	0.112
H15	0.065
H16	0.093
H17	0.074
H18	0.0884
H19	0.116
H20	0.066
H21	0.120
H22	0.067
H23	0.060
H24	0.147
H25	0.114
H26	0.064
H27	0.077
H28	0.058
H29	0.121
H30	0.083
H31	0.077
Li32	0.908

Section S5. Supplementary References

1. Lu T, Chen F. Multiwfn: A multifunctional wavefunction analyzer. *J Comput Chem* 2012; **33**: 580-592.

2. Wang J, Wolf RM, Caldwell JW *et al.* Development and testing of a general amber force field. *J Comput Chem* 2004; **25**: 1157-1174.

3. Molinari N, Mailoa JP, Kozinsky B. Effect of Salt Concentration on Ion Clustering and Transport in Polymer Solid Electrolytes: A Molecular Dynamics Study of PEO–LiTFSI. *Chem Mater* 2018; **30**: 6298-6306.
